# DFsn collaborates with Highwire to down-regulate the Wallenda/DLK kinase and restrain synaptic terminal growth

**DOI:** 10.1186/1749-8104-2-16

**Published:** 2007-08-15

**Authors:** Chunlai Wu, Richard W Daniels, Aaron DiAntonio

**Affiliations:** 1 Department of Molecular Biology and Pharmacology, Washington University School of Medicine, St. Louis, MO 63110, USA

## Abstract

**Background:**

The growth of new synapses shapes the initial formation and subsequent rearrangement of neural circuitry. Genetic studies have demonstrated that the ubiquitin ligase Highwire restrains synaptic terminal growth by down-regulating the MAP kinase kinase kinase Wallenda/dual leucine zipper kinase (DLK). To investigate the mechanism of Highwire action, we have identified DFsn as a binding partner of Highwire and characterized the roles of DFsn in synapse development, synaptic transmission, and the regulation of Wallenda/DLK kinase abundance.

**Results:**

We identified DFsn as an F-box protein that binds to the RING-domain ubiquitin ligase Highwire and that can localize to the *Drosophila *neuromuscular junction. Loss-of-function mutants for *DFsn *have a phenotype that is very similar to *highwire *mutants – there is a dramatic overgrowth of synaptic termini, with a large increase in the number of synaptic boutons and branches. In addition, synaptic transmission is impaired in *DFsn *mutants. Genetic interactions between *DFsn *and *highwire *mutants indicate that DFsn and Highwire collaborate to restrain synaptic terminal growth. Finally, DFsn regulates the levels of the Wallenda/DLK kinase, and *wallenda *is necessary for *DFsn*-dependent synaptic terminal overgrowth.

**Conclusion:**

The F-box protein DFsn binds the ubiquitin ligase Highwire and is required to down-regulate the levels of the Wallenda/DLK kinase and restrain synaptic terminal growth. We propose that DFsn and Highwire participate in an evolutionarily conserved ubiquitin ligase complex whose substrates regulate the structure and function of synapses.

## Background

The connectivity and functionality of a neural circuit depends on the structure of its constituent neurons' presynaptic fields. Different neurons make very different synaptic trees – a serotonergic neuron will synapse with thousands of neurons throughout the brain, while climbing fibers from the medulla may synapse with only a single Purkinje cell in the cerebellum. Individual neurons with the same identity can also have different sized synaptic arbors and this has functional consequences: during synaptic competition at the vertebrate neuromuscular junction (NMJ), motoneurons with larger arbors are at a disadvantage when competing against those with smaller arbors [1]. Molecular mechanisms that control the size and structure of a neuron's presynaptic field are key regulators of the development, function, and plasticity of neural circuits.

In *Drosophila*, genetic studies have identified a number of signaling pathways that regulate the morphology of the presynaptic arbor made by motoneurons onto muscles (reviewed in [2]). One such pathway requires *highwire *– in *highwire *mutants there is a dramatic increase in the complexity of the synaptic terminal, with a large increase in the number of synaptic boutons and branches [3]. This function for *highwire *is evolutionarily conserved: studies in worms, fish, and mammals all suggest that *highwire *homologs are required for normal synaptic development. In *Caenorhabditis elegans*, the homolog *rpm-1 *regulates the number, spacing, and morphology of presynaptic active zones [4,5], mutations in the zebrafish homolog *esrom *disrupt retinotectal projections [6], while mice carrying large deletions that remove the murine homolog *phr1 *and adjacent genes disrupt NMJ morphology [7].

Highwire and its homologs are huge proteins on the order of 5,000 amino acids that share a series of domains that likely perform distinct functions. These include an amino-terminal Ran GTPase exchange factor domain that can inhibit adenylate cyclase [8], two Phr repeats of unknown function, a Myc-binding domain [9], and a carboxy-terminal RING domain that can function as an E3 ubiquitin ligase. Numerous studies demonstrate a central role for this ubiquitin ligase activity in the molecular mechanism of Highwire action. In both *C. elegans *and zebrafish, the isolated RING domain can promote ubiquitination *in vitro *[6,10], and mutations that disrupt the RING finger abolish function [6,11]. In addition, overexpression of ubiquitin hydrolases enhances the *highwire *phenotype and promotes synaptic terminal overgrowth at the *Drosophila *NMJ [12].

What are the key targets of Highwire regulation? As befits a large, multi-domain protein, Highwire and its homologs interact with many candidate targets, including the co-SMAD Medea, adenylate cyclases, members of the tuberous sclerosis (TSC) complex, and the oncogenic protein Myc [8,9,13,14]. One particularly compelling candidate is the mitogen-activated protein (MAP) kinase kinase kinase (MAPKKK) Wallenda in *Drosophila *and its *C. elegans *homologue dual leucine zipper kinase (DLK) [10,15]. In both worms and flies, levels of the Wallenda/DLK kinase are increased in the absence of *highwire*/*rpm-1*, the synaptic defects of *highwire*/*rpm-1 *mutants are suppressed by mutations in either Wallenda/DLK or their downstream MAP kinases, and increasing the levels of Wallenda/DLK is sufficient to phenocopy the synaptic defects of *highwire*/*rpm-1 *mutants. In *C. elegans*, DLK is required for some but not all *rpm-1 *phenotypes, indicating that *rpm-1 *may have other targets. In *Drosophila*, *wallenda *is necessary for all of the synaptic morphology defects in a *highwire *mutant, but it is not required for *highwire*-dependent regulation of synaptic function [15]. Since the regulation of synaptic transmission by *highwire *requires a functional ubiquitin ligase domain [11], Highwire very likely targets at least one protein in addition to Wallenda.

RING finger E3 ubiquitin ligases can function to promote ubiquitination of target proteins on their own or as members of large E3 protein complexes [16]. In *C. elegans*, RPM-1 binds to Cullin-1, Skp-1, and the F-box protein FSN-1, leading to the hypothesis that RPM-1 functions as part of an atypical SCF (Skp1-cullin-F box protein) E3 ubiquitin ligase complex [17]. F-box proteins such as FSN-1 determine the substrate specificity of the E3 ligase complex [18]. Mutations in *C. elegans fsn-1 *lead to many of the same phenotypes as *rpm-1 *mutants, consistent with the hypothesis that they function together to regulate synapse development. However, genetic and biochemical data indicate that the receptor tyrosine kinase anaplastic lymphoma kinase (ALK) is the functionally relevant target of FSN-1, since levels of ALK increase in an *fsn-1 *mutant and the *fsn-1 *phenotype is suppressed by mutations in ALK [17]. However, *rpm-1 *mutants are suppressed much more strongly by DLK than by ALK [10], so *rpm-1 *and *fsn-1 *may target different substrates. In fact, it is not known if FSN-1 biochemically or genetically interacts with DLK. This presents a paradox – two components of the same E3 ligase work together to regulate synaptic development, but apparently via different molecular pathways.

These findings in *C. elegans *raise a number of questions. First, is the binding between FSN-1 and RPM-1/Highwire conserved in *Drosophila*? Since the *C. elegans *genome encodes more than 300 F-box proteins and *Drosophila *encodes only 33, this is not a given [18,19]. Second, does Fsn regulate synaptic terminal growth and synaptic function in *Drosophila*? Third, does Fsn collaborate with Highwire to regulate the levels of the Wallenda/DLK kinase and is this necessary for normal synaptic development? Fourth, does the regulation of other Highwire targets require collaboration with Fsn, or does Highwire function alone or with other F-box proteins? We identified the *Drosophila *homolog of FSN-1, DFsn, in a proteomic screen as a Highwire-binding protein. We have addressed the questions above by undertaking a genetic analysis of *DFsn *function in *Drosophila*. Our results demonstrate that DFsn collaborates with Highwire to restrain synaptic terminal growth by down-regulating the same target, the Wallenda/DLK kinase.

## Results

### *Drosophila *Fsn is a Highwire-interacting protein

**Figure 1:**
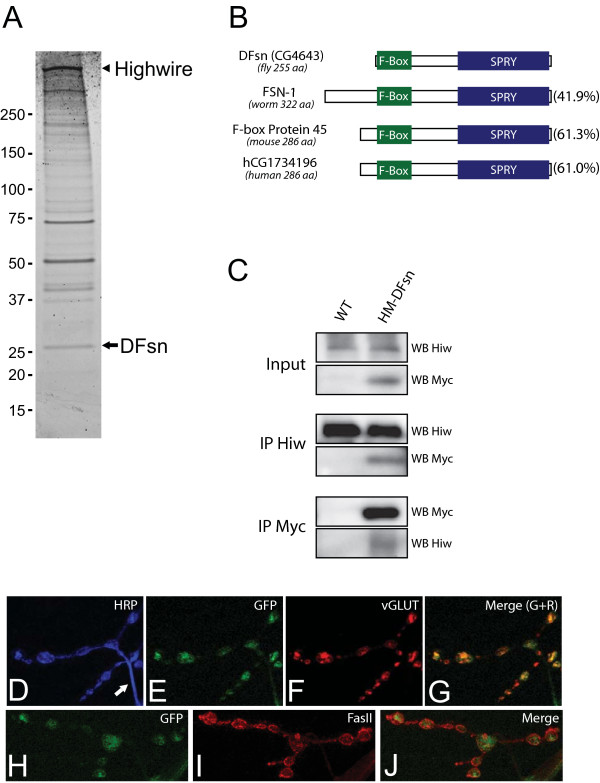
DFsn is identified as a Highwire-interacting protein. **(a) **The Highwire-associated complex was purified by the TAP procedure (see Materials and methods) and analyzed by one-dimensional SDS-PAGE gel followed by Sypro Ruby staining. A representative gel is shown and full-length Highwire is marked with an arrowhead. Mass spectrometry identified one of the Highwire-interacting proteins as DFsn (approximately 27 kDa, indicated by arrow). **(b) **Schematic of DFsn protein (CG4643 in *Drosophila*) and its homologs in *C. elegans*, mouse and human. Green boxes denote the F-Box and blue boxes denote the SPRY domain. The overall amino acid identity to the DFsn protein is reported in the parenthesis on the right. **(c) **Larval brain lysate of either wild type (WT) flies or flies neuronally expressing His-Myc-tagged DFsn (HM-DFsn) was subject to co-immunoprecipitation (IP) with either anti-Highwire antibody (Hiw2b) or anti-Myc antibody (9E10). Both the input and the IP complexes were analyzed by western blots (WB) with anti-Myc or anti-Highwire antibody. **(d-j) **When expressed in the central nervous system, GFP-DFsn is localized to NMJs. The localization of GFP-DFsn at the synapses is analyzed by double staining with anti-GFP and synaptic vesicle marker DVGLUT (e-g), or periactive zone marker FasII (h-j). In (d), the presynaptic terminal of (e-g) was visualized by HRP (horseradish peroxidase) staining; the arrow indicates a preterminal axon.

In an effort to understand the mechanism of action of Highwire, we have identified Highwire-binding proteins from the *Drosophila *nervous system. Tandem affinity purification (TAP) is a powerful two-step purification scheme for isolating a protein of interest and its binding partners [20]. We generated transgenic flies expressing a TAP-tagged Highwire and demonstrated that this fusion protein is functional since it rescues the *highwire *loss-of-function phenotypes [11]. We also generated a transgene encoding TAP-tagged HighwireΔRING (HiwΔRING), which carries two Cys-to-Ser mutations in the conserved RING domain, and demonstrated that HiwΔRING acts as a potent dominant negative when expressed in neurons [11]. Both *highwire *transgenes were tested in our TAP system. We found that HiwΔRING shows a much stronger enrichment in the final complex than Highwire, possibly because of a higher stability of HiwΔRING (data not shown). Thus, we expressed TAP-HiwΔRING in the *Drosophila *central nervous system (CNS), purified full-length HiwΔRING and associated proteins (see Materials and methods for details), and analyzed the purified complex by two independent approaches. First, the eluted proteins were resolved on SDS-PAGE and the sypro-stained protein bands were excised, digested with trypsin, and subjected to liquid chromatography-tandem mass spectrometry (LC-MS/MS). The searching program MASCOT was used to identify the protein species. Peptide mass fingerprinting and MS/MS peptide sequencing (data not shown) identified the bait protein Highwire as the 600 kDa band in Figure 1a. In addition, CG4643 was identified as the 27 kDa band indicated in Figure 1a based on the recovery of two unique peptides (SDLLASVSTYK, NVYIKPNGFTLHR) that are present in CG4643. Second, LC-MS/MS was used to directly analyze the purified protein complex without an intermediate SDS-PAGE separation. Again, in addition to Highwire peptides, five unique peptides from CG4643 were recovered (ESLKSDLLASVSTYK, NPVAQSTDAAR, YQVGER, IRVILDCEDNTLSFEK, NYEFLGVAFR).

CG4643 is predicted to encode a 27 kDa protein containing an F-box domain, a WD-40 repeat, and a spla and ryanodine receptor (SPRY) domain. Its closest homolog in *C. elegans *is FSN-1, which is composed of the same functional domains and which binds to the RPM-1, the *C. elegans *homolog of Highwire. The two proteins share 41.9% homology, and define a small sub-family of F-box proteins with SPRY domains of which there is only a single member in worms, flies, mice (F-box protein 45 (GenBank:AAH26799)), and humans (hCG1734196 (GenBank:EAW53643)) with high homology across species (Figure 1b). Based on the sequence similarity of CG4643 and FSN-1, we have renamed CG4643 *DFsn*, for *Drosophila *Fsn.

The fact that TAP-HiwΔRING can pull down DFsn indicates that an intact RING domain is not required for the Highwire-DFsn interaction. To test whether wild-type Highwire also interacts with DFsn, we performed the following co-immunoprecipitation experiments. Since we have been unable to generate an antibody that recognizes endogenous DFsn, we generated transgenic flies that express His-Myc-tagged DFsn. As described below, the transgene encoding this is functional since it can rescue a *DFsn *mutant. We expressed His-Myc-tagged DFsn throughout the nervous system and performed immunoprecipitations from third instar larvae brains. Immunoprecipitations (IPs) and western blot analysis was performed with either affinity-purified polyclonal antibodies raised against Highwire [11] or a monoclonal antibody to Myc. As shown in Figure 1c, Myc-DFsn is present when endogenous Highwire is immunoprecipitated. Conversely, endogenous Highwire is detected in the IP fraction when Myc-DFsn is immunoprecipitated by the anti-Myc antibody. In addition, neither endogenous Highwire nor Myc-DFsn is detected when a control protein, *Drosophila *vesicular Glutamate transporter (DVGLUT), is immunoprecipitated from Myc-DFsn expressing larval brains (data not shown). These data demonstrate that endogenous Highwire physically interacts with DFsn. While eukaryotic genomes can express hundreds of F-box proteins and a similar number of RING domain ubiquitin ligases [18,21], these data and the previous work of Liao *et al*. [17] demonstrate that the physical interaction between Highwire/RPM-1 and Fsn is evolutionarily conserved.

Since our efforts to generate an antibody against DFsn were unsuccessful, we generated a functional green fluorescent protein (GFP)-tagged *DFsn *transgene. When the transgene is expressed by a pan-neuronal Gal4 driver, GFP-DFsn localizes to the NMJ (Figure 1e,h). Double staining with markers for synaptic vesicles (Figure 1e–g) and periactive zones (Figure 1h–j) demonstrates that GFP-DFsn is not restricted to any of these compartments. GFP-DFsn is also detectable in the axon and neuronal cell body (data not shown), although the staining of GFP-DFsn is much stronger in synaptic boutons than in the preterminal axon (Figure 1e, arrow), suggesting a synaptic enrichment. GFP-Highwire encoded by a pan-neuronally expressed transgene has a very similar localization pattern [11]. These data are consistent with the presence of a Highwire-DFsn complex at the NMJ.

### DFsn is required to restrain synaptic terminal growth at the NMJ

**Figure 2:**
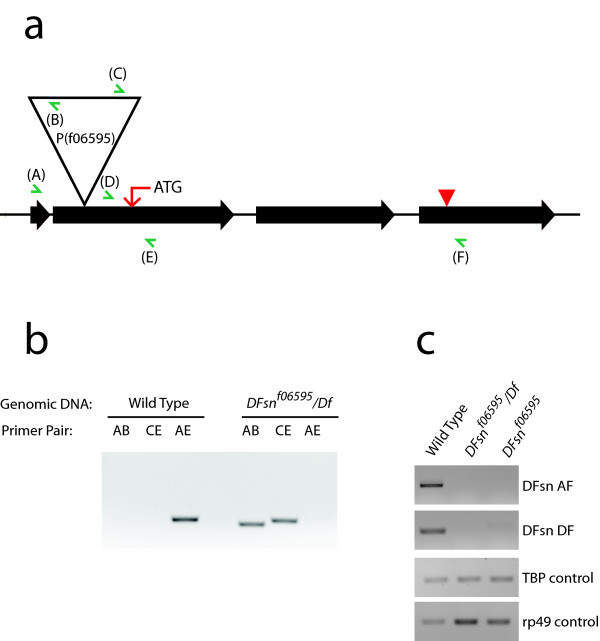
A transposable-element-insertion mutant of *DFsn*. **(a) **The gene structure of *DFsn*. The black-arrowed boxes indicate exons. A piggyBac transposable element, P(f06595) (represented by a big triangle), inserts in the second exon, 121 base-pairs upstream of the start codon (indicated by the red arrow). The red arrowhead points to the stop codon. Green arrows mark forward or reverse primers used in the PCR experiments described in (b, c). **(b) **PCR reactions using the indicated primer pairs and genomic DNA of wild-type or *DFsn*^*f06595*^*/Df *[*P(f06595)*/*Df(2R)7872*] flies demonstrate the location of the P-element insertion. **(c) **RT-PCRs were performed using total RNAs from wild-type, *DFsn*^*f06595*^/*Df *or *DFsn*^*f06595 *^[*P(f06595)/P(f06595)*] flies and poly dT oligo primer for the reverse transcription reactions. PCR performed with the indicated primer pairs at 26 cycles are shown here. The normal transcript in the wild type (accessed by primer pairs AF and DF) is disrupted in the *DFsn *mutants. Additional cycles of PCR only revealed mis-spliced *DFsn *transcripts in the *DFsn *mutants. Transcripts of TBP (TATA box binding protein) and rp49 (ribosomal protein 49) are analyzed as internal control.

**Figure 3:**
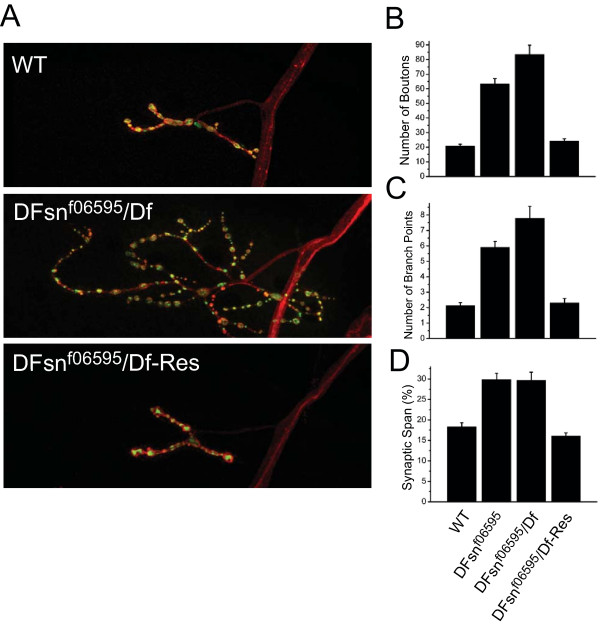
DFsn is required to restrain synaptic terminal growth at the NMJ. **(a) **Representative confocal images of muscle 4 synapses co-stained with DVGLUT (green) and FasII (red), in wild-type (WT), and *DFsn*^*f06595*^*/Df-Res *[presynaptic rescue: *P(f06595)/Df(2R)7872; elav Gal4/UAS-GFP-DFsn*] third instar larvae. **(b-d) **Quantification of bouton number (b), number of branch points (c) and synaptic span (d) in WT, *DFsn*^*f06595 *^[*P(f06595)/P(f06595)*], *DFsn*^*f06595*^*/Df *and *DFsn*^*f06595*^*/Df-Res *third instar larvae; n = 36, 21, 23 and 29 cells, respectively. Both *DFsn*^*f06595 *^and *DFsn*^*f06595*^*/Df *are significantly different from WT for all parameters measured (*p *< 0.001), while *DFsn*^*f06595*^*/Df *shows a more severe phenotype in number of boutons and number of branching points than *DFsn*^*f06595 *^(*p *< 0.05). The morphological defects in *DFsn*^*f06595*^*/Df *are rescued by presynaptic expression of GFP-DFsn in all parameters measured (*p *< 0.001 for *DFsn*^*f06595*^*/Df-Res *versus *DFsn*^*f06595*^*/Df*; *p *> 0.5 for *DFsn*^*f06595*^*/Df-Res *versus WT).

*C. elegans *FSN-1 is required for normal presynaptic differentiation [17], and its phenotype partially overlaps with the *rpm-1 *phenotype. Having shown that the Highwire-DFsn interaction is conserved in *Drosophila*, we wished to test whether DFsn is also required for normal synapse development. We obtained a chromosomal deficiency, Df(2R)7872, that removes *DFsn *and flanking genes, as well as a transposable-element-insertion mutant in the *DFsn *locus from the *Drosophila *stock center. The insert, P(f06595), is a piggyBac P-element inserted in the second exon of the *DFsn*, 121 base-pairs upstream of the start codon (Figure 2a,b). RT-PCR data demonstrate that transcription of *DFsn *is disrupted in homozygous f06595 and hemizygous P(f06595)/Df(2R)7872 mutants (Figure 2c). Both the homozygous and hemizygous adult *DFsn *mutants are viable and male-sterile. Morphological analysis of the NMJ from P(f06595)/P(f06595) and P(f06595)/Df(2R)7872 larvae reveals dramatic synaptic terminal overgrowth (Figure 3). We quantified this phenotype at the NMJ of a single identified motoneuron, MN4-1b [22], which forms the type Ib innervation onto muscle 4, but all phenotypes are qualitatively similar at every type I NMJ, including the muscle 6/7 NMJ (Additional file 1). We quantified three metrics of synaptic structure: bouton number, number of branch points and synaptic span (the extent of the muscle covered by the synapse). The P(f06595)/Df(2R)7872 mutant shows an almost four-fold increase in both bouton number and number of branch points when compared with the wild-type NMJ (Figure 3). The synaptic span of the P(f06595)/Df(2R)7872 mutant is also increased from 18% in wild type to 30% (Figure 3). This synaptic terminal overgrowth is quantitatively and qualitatively very similar to that observed in a *highwire *null mutant [11]. The homozygous P(f06595)/P(f06595) mutant also has a large increase in bouton number, branch point number, and synaptic span, but the phenotype is less severe than in the P(f06595)/Df(2R)7872 mutant. Since the mutant phenotype of P(f06595) is enhanced when placed over a deletion, P(f06595) is behaving as a genetic hypomorph.

Highwire regulates synaptic terminal growth presynaptically. If DFsn functions with Highwire to restrain this growth, then it should also be required in the presynaptic motoneuron. We generated transgenic flies capable of expressing *DFsn *in a tissue-specific manner (*UAS-GFP-DFsn*). We expressed GFP-DFsn in neurons with elav-Gal4, a pan-neural Gal4 driver, in the *DFsn *mutant background [*P*(*f06595)/Df7872; UAS-GFP-DFsn/elav Gal4*]. To assess rescue, we again quantified bouton number, synaptic branching, and synaptic span at the MN4-Ib NMJ onto muscle 4. All of these aspects of synaptic terminal overgrowth are rescued by the presynaptic expression of *DFsn *(Figure 3). The presence of the elav-Gal4 transgene without the *UAS-GFP-DFsn *transgene fails to rescue the *DFsn *mutant (*p *> 0.4 for number of boutons and branch points). Furthermore, synaptic terminal overgrowth of the *DFsn *mutant is also fully rescued when GFP-DFsn is expressed selectively in motoneurons (driven by D42; Additional file 1), indicating *DFsn *functions cell autonomously in the motoneuron to restrain synaptic terminal growth. Although we only quantified NMJs onto muscle 4, all type I NMJs, including those on muscle 6/7, show qualitatively similar rescue (Additional file 1). The transgene encoding the His-Myc-tagged DFsn also rescued the mutant phenotype (data not shown), so the fusion proteins used for immunoprecipitation and immunolocalization above are both functional. These data demonstrate that *DFsn *is responsible for the phenotype of the P(f06595) mutant, which we have renamed *DFsn*^*f06595*^. These data demonstrate that *DFsn *is required in the presynaptic motoneuron to restrain synaptic terminal growth at the *Drosophila *NMJ.

### *Drosophila Fsn *is required for normal synaptic function

**Figure 4:**
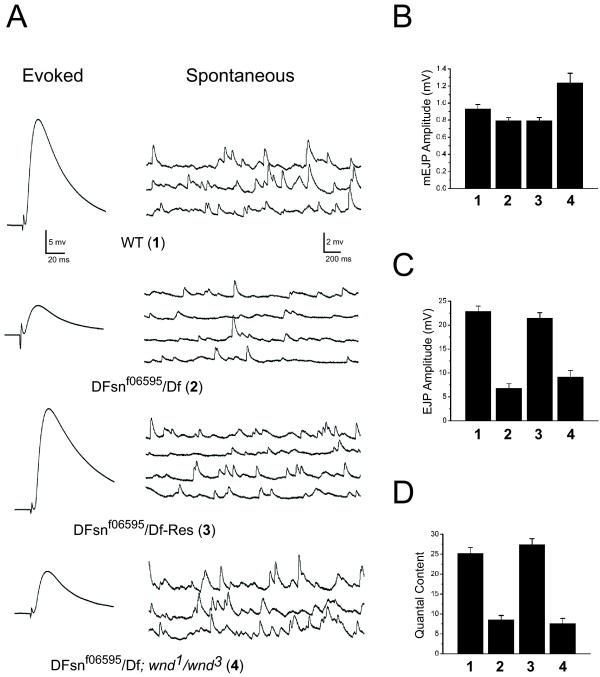
DFsn is required for normal synaptic function. **(a) **Representative traces of evoked and spontaneous transmitter release recorded from wild-type (WT), *DFsn*^*f06595*^*/Df *[*P(f06595)/Df(2R)7872*], *DFsn*^*f06595*^*/Df-Res *[presynaptic rescue: *P(f06595)/Df(2R)7872; elav Gal4/UAS-HM-DFsn*] and *DFsnf06595/Df; wnd*^1^*/wnd*^3 ^third instar larvae. **(b-e) **The mean mEJP amplitude (b), EJP amplitude (c) and quantal content (d) are plotted for WT, *DFsn*^*f06595*^*/Df*, *DFsn*^*f06595*^*/Df-Res *and *DFsnf06595/Df; wnd*^1^*/wnd*^3 ^(n = 13, 15, 10 and 9 cells, respectively). The *DFsn*^*f06595*^*/Df *mutant shows reduced EJP amplitude and reduced quantal content (*p *< 0.001 for *DFsn*^*f06595*^*/Df *versus WT). These electrophysiological defects in the *DFsn*^*f06595*^*/Df *mutant are all rescued by presynaptic expression of DFsn (*p *< 0.001 for *DFsn*^*f06595*^*/Df-Res *versus *DFsn*^*f06595*^*/Df*;*p *> 0.5 for *DFsn*^*f06595*^*/Df-Res *versus WT). *wallenda *suppresses the reduction of mini frequency in the *DFsn*^*f06595*^*/Df *mutant (*p *> 0.5 for *DFsn*^*f06595*^*/Df; wnd*^1^*/wnd*^3 ^versus WT). The EJP amplitude and the quantal content of the double mutant *DFsnf06595/Df; wnd*^1^*/wnd*^3 ^are not significantly different than *DFsn*^*f06595*^*/Df *(*p *> 0.1), demonstrating that *wallenda *does not suppress the *DFsn*^*f06595*^*/Df *defect in evoked release.

Highwire is not only required to restrain synaptic terminal growth, but also to promote synaptic function. Both of these activities require the ligase function of Highwire, but Highwire regulates distinct molecular pathways to affect synapse morphology and function. Since F-box proteins can confer specificity to ubiquitin ligases, we wondered whether *DFsn *is also required to regulate synaptic release. We analyzed synaptic transmission in *DFsn *mutants by performing intracellular recordings from muscle 6 (segments A3 and A4) of third instar larvae and measured both spontaneous and evoked neurotransmitter release (Figure 4). Quantal size (mEJP amplitude), the postsynaptic response to the spontaneous fusion of a single synaptic vesicle, is unchanged in *DFsn *mutants (Figure 4b). However, evoked release is greatly reduced – *DFsn *mutant larvae have a 70% reduction (*p *< 0.001) in the excitatory junctional potential (EJP) compared with wild type (Figure 4c). The reduction in evoked release is due to a 66% decrease (*p *< 0.001) in quantal content, the number of vesicles released by a nerve following an action potential, in the mutant (Figure 4d). Presynaptic expression of the wild-type *UAS-DFsn *transgene rescues the deficit in both evoked neurotransmitter release and quantal content (Figure 4), indicating DFsn functions presynaptically to regulate synaptic function. The decrease in evoked synaptic release observed in the *DFsn *mutant is very similar to that seen in *highwire *null mutants, although the hypomorphic *DFsn *mutant does not show the same modest decrease in quantal size observed in the *hiw *null mutant [11].

### *DFsn *genetically interacts with *highwire *to restrain synaptic terminal growth

**Figure 5:**
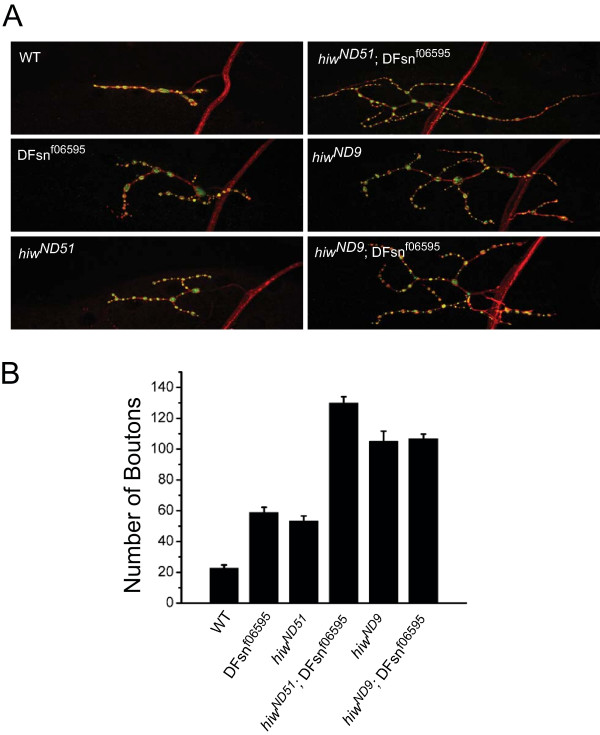
DFsn genetically interacts with Highwire to restrain synaptic terminal growth. **(a) **Representative confocal images of muscle 4 synapses co-stained with DVGLUT (green) and FasII (red), in wild-type (WT), *DFsn*^*f06595*^, *hiw*^*ND*51^, *hiw*^*ND*51^; *DFsn*^*f06595*^, *hiw*^*ND*9 ^and *hiw*^*ND*9^; *DFsn*^*f06595 *^third instar larvae. **(b) **Quantification of bouton number of muscle 4 synapses in WT, *DFsn*^*f06595*^, *hiw*^*ND*51^, *hiw*^*ND*51^; *DFsn*^*f06595*^, *hiw*^*ND*9 ^and *hiw*^*ND*9^; *DFsn*^*f06595 *^third instar larvae (n = 27, 34, 27, 28, 22, and 39 cells, respectively). The number of boutons in the *hiw*^*ND*51^; *DFsn*^*f06595 *^double mutant is increased compared to *hiw*^*ND*51 ^(*p *< 0.001). The number of boutons in the *hiw*^*ND*9^; *DFsn*^*f06595 *^double mutant is not significantly different from *hiw*^*ND*9 ^(*p *> 0.5), demonstrating a lack of enhancement of the *hiw*^*ND*9 ^phenotype by *DFsn*^*f06595*^.

The analysis above demonstrates that *DFsn *and *highwire *loss-of-function mutants have very similar morphological and physiological phenotypes. Since they physically interact, these similar phenotypes suggest that DFsn and Highwire function together in a complex. To test this hypothesis, we analyzed genetic interactions between *DFsn *and *highwire*. If DFsn and Highwire function together in a complex, then we predict that hypomorphic (partial loss-of-function) alleles of *DFsn *should enhance the phenotype of a hypomorphic *highwire *mutant. We generated double mutants between the hypomorphic *DFsn*^*f06595 *^mutant and the weak *highwire *allele *hiw*^*ND*51^, which carries an amino acid change at residue 2054 (G2054R) [3]. While *hiw*^*ND*51 ^and *DFsn*^*f06595 *^mutants each show mild synaptic terminal overgrowth compared to wild type, the double mutant shows dramatically overgrown synapses (Figure 5). In addition to the large increase in bouton number (Figure 5b), there is also enhancement of synaptic branching, with the number of branch points being: wild type, 2.2 ± 0.3 (n = 27); *hiw*^*ND*51^, 4.3 ± 0.3 (n = 27); *DFsn*^*f06595*^, 6.5 ± 0.4 (n = 34); *hiw*^*ND*51^, *DFsn*^*f06595*^, 10.8 ± 0.6 (n = 28). The enhancement of a weak *hiw *allele by the *DFsn *hypomorph is consistent with the two proteins acting in a complex; however, it is also consistent with the two proteins acting in parallel pathways. To distinguish between these possibilities, we generated double mutants between the *DFsn*^*f06595 *^mutant and *hiw*^*ND*9^, a genetic null for *highwire *harboring a premature stop. If DFsn and Highwire function together, then loss of DFsn should not enhance a *highwire *null, since it is already without function. However, if the proteins work in parallel pathways, then the *DFsn *phenotype would be additive and it should enhance either a hypomorphic or null *highwire *allele. We found that *DFsn*^*f06595 *^does not enhance *hiw*^*ND*9^. Instead, *hiw*^*ND*9 ^and the *hiw*^*ND*9^, *DFsn*^*f06595 *^double mutant have a very similar synaptic terminal overgrowth phenotype (Figure 5). The double mutant also fails to enhance the number of synaptic branch points, with the number of branch points being: *hiw*^*ND*9^, 9.6 ± 0.8 (n = 22); *hiw*^*ND*9^, *DFsn*^*f06595*^, 7.4 ± 0.4 (n = 39). Synaptic terminal growth is not saturated in a strong *highwire *mutant [13], so the absence of enhancement by the addition of a *DFsn *mutant is strong genetic evidence that DFsn and Highwire collaborate to restrain synaptic terminal growth.

### DFsn down-regulates the levels of the Wallenda/DLK kinase to restrain synaptic terminal growth

How do Highwire and DFsn regulate synaptic terminal growth? In both fly and worm, Highwire/RPM-1 down-regulates the protein levels of the Wallenda/DLK kinase. Increased levels of Wallenda/DLK over-activate downstream MAP kinase cascades and lead to altered synapse development. Mutations in *wallenda*/*DLK *suppress the *highwire*/*rpm-1 *phenotypes. However, in worm, FSN-1 regulates the levels of the tyrosine kinase ALK and ALK mutants suppress the *fsn-1 *phenotype, leading to the suggestion that FSN-1 regulates synapse development by down-regulating ALK [17]. If Highwire and DFsn collaborate as part of a ubiquitin ligase complex, then we predict they should both regulate the same target protein. We have found that ALK is postsynaptic at the *Drosophila *NMJ (AD, unpublished observation), so we have focused our attention on Wallenda.

**Figure 6:**
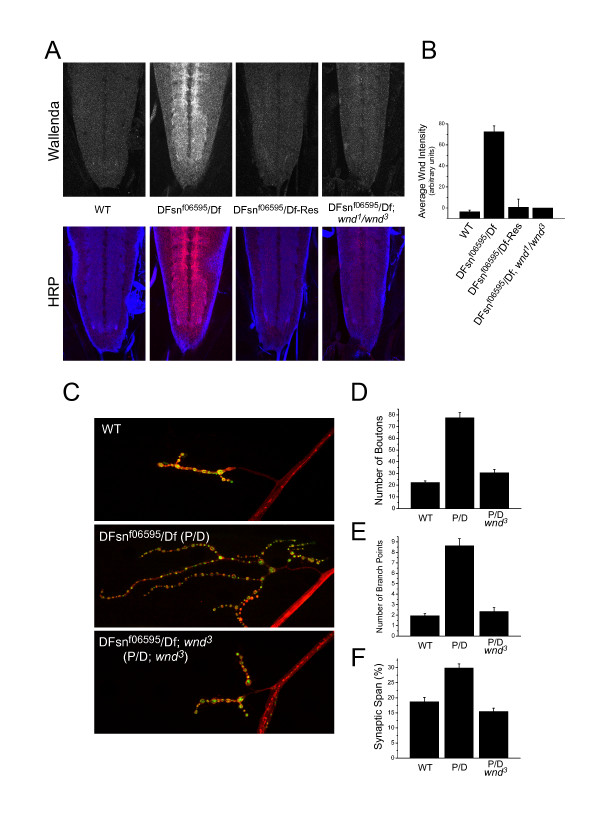
DFsn down-regulates the levels of the Wallenda/DLK kinase to restrain synaptic terminal growth. **(a) **Staining for endogenous Wallenda in central nervous system neuropil in ventral nerve cords of wild-type (WT), *DFsn*^*f06595*^*/Df *[*P(f06595)/Df(2R)7872*], *DFsn*^*f06595*^*/Df-Res *[presynaptic rescue: *P(f06595)/Df(2R)7872; elav Gal4/UAS-GFP-DFsn*] and *DFsnf06595/Df; wnd*^1^*/wnd*^3^ third-instar larvae. Upper panels show Wallenda staining alone, and lower panels show co-staining of Wallenda (red) with the synaptic marker HRP (horseradish peroxidase) (blue). **(b) **Quantification of average Wallenda staining in the neuropil of third-instar larvae for the genotypes shown in (a). The Wallenda intensity was measured for each genotype and the background intensity measured for *DFsnf06595/Df; wnd*^1^*/wnd*^3 ^was subtracted. Wallenda staining in the *DFsn*^*f06595*^*/Df *mutant was significantly higher than WT (*p *< 0.001). Presynaptic expression of a *GFP-DFsn *transgene rescues the increase of Wallenda level (*p *> 0.5 for *DFsn*^*f06595*^*/Df-Res *versus WT). **(c) **Representative confocal images of muscle 4 synapses co-stained with DVGLUT (green) and FasII (red), in WT, *DFsn*^*f06595*^*/Df *and *DFsn*^*f06595*^*/Df; wnd*^3 ^third instar larvae. **(d-f) **Quantification is shown for bouton number (d) and synaptic branch point number (e) and synaptic span (f) for WT, *DFsn*^*f06595*^*/Df *and *DFsn*^*f06595*^*/Df; wnd*^3 ^(n = 23, 23, and 22 cells, respectively). The *wallenda *mutant fully suppresses the morphological defects in the *DFsn*^*f06595*^*/Df *mutant (*p *> 0.1 for *DFsn*^*f06595*^*/Df*; *wnd*^3 ^versus WT in number of boutons and branch points; *p *< 0.001 for *DFsn*^*f06595*^*/Df; wnd*^3 ^ versus *DFsn*^*f06595*^*/Df *in synaptic span, number of boutons and branch points).

We first tested if DFsn, like Highwire, regulates the levels of Wallenda in the nervous system. Wallenda protein is undetectable in the nerve cord of wild-type larvae, but is abundant in the synaptic neuropil of ventral nerve cords from *DFsn *mutants (Figure 6a,b). This staining is due to Wallenda protein since it is absent in *DFsn, wnd *double mutant animals. Expression of a *DFsn *transgene rescues the increase in Wallenda levels in the *DFsn *mutant (Figure 6a,b). Hence, DFsn, like Highwire, is required to down-regulate the levels of the Wallenda/DLK kinase. While Highwire and DFsn are each necessary to down-regulate Wallenda/DLK, their simultaneous over-expression in neurons is not sufficient to reduce the level of Wallenda protein (data not shown), indicating that the Hiw-DFsn ubiquitin ligase complex likely includes other components.

Having demonstrated that DFsn regulates Wallenda levels, we tested whether Wallenda is required for synaptic terminal overgrowth in the *DFsn *mutant. As shown in Figure 6c–f, loss of *wallenda *suppresses the synaptic morphology defects of the *DFsn *mutant, restoring bouton number, branching number, and synaptic span to near wild-type levels. Therefore, Wallenda is necessary for synaptic terminal overgrowth in both the *DFsn *and *highwire *mutants. While *wnd *mutants suppress the morphological defects of the *DFsn *mutant, they do not suppress the electrophysiological phenotype. Quantal content is not significantly different between *DFsn *single mutants and *DFsn, wnd *double mutants, while both show impaired release compared to wild type (*p *< 0.001; Figure 4d). Therefore, DFsn, like Highwire, must regulate at least two pathways. In both mutants, the down-regulation of Wallenda is necessary to restrain synaptic terminal growth, while a genetically separable pathway is involved in promoting synaptic release.

## Discussion

Highwire and RPM-1 act as ubiquitin ligases to regulate synaptic development. Liao *et al*. [17] have proposed that, in *C. elegans*, RPM-1 participates in an atypical SCF ubiquitin ligase complex with the F-box protein FSN-1. Consistent with this hypothesis, RPM-1 binds to FSN-1 as well as to Skp-1 and Cullin-1, core components of SCF complexes. In addition, FSN-1 null mutants have very similar phenotypes to *rpm-1 *mutants at GABAergic synapses, but weaker phenotypes in DD motoneurons and sensory neurons. The difference in phenotypes suggests that RPM-1 interacts with other F-box proteins in addition to FSN-1, acts as a ubiquitin ligase without an F-box partner, or has ubiquitin-independent functions. The target of the RPM-1/FSN-1 complex in *C. elegans *is not clear. Biochemical and genetic data indicate that the receptor tyrosine kinase ALK is the functionally relevant target for FSN-1 [17], while the MAPKKK DLK is the functionally relevant target for RPM-1 [10]. Our data in *Drosophila *support the model from worms that RPM-1 and FSN-1 form a functional ubiquitin ligase complex, but simplify the model by demonstrating that in *Drosophila *both components target the same substrate.

We demonstrate that Highwire binds the *Drosophila *homolog of FSN-1, DFsn. Therefore, the physical association of Highwire/RPM-1 and DFsn/FSN-1 is evolutionarily conserved. While eukaryotic genomes can encode hundreds of F-box proteins like DFsn, in worms, flies, mice, and humans there is only a single F-box protein that also contains an SPRY domain, and each is more closely related by sequence to each other than to other F-box proteins. Since the binding of DFsn/FSN-1 to Highwire/RPM-1 is conserved, we speculate that the mouse and human homologs of DFsn, F-box protein 45 and hCG1734196, will bind to and function with Phr [23] and PAM [9], the mouse and human homologs of Highwire, respectively. Indeed, expression analysis demonstrates that both the F-box protein 45 and Phr are expressed in a very similar pattern in the mouse brain [24].

Our results suggest that the interaction of Highwire with DFsn is required for Highwire activity. Loss-of-function mutants for *highwire *and *DFsn *have qualitatively and quantitatively similar phenotypes – both are required to restrain synaptic terminal growth and promote synaptic release. Both Highwire and DFsn are necessary to down-regulate the levels of the MAPKKK Wallenda/DLK, and *wallenda *mutants suppress the morphological but not physiological phenotypes of both *highwire *and *DFsn*. Finally, genetic data support the model that Highwire and DFsn function together during synaptic development – *DFsn *mutants enhance the phenotype of a *highwire *hypomorph but not of a *highwire *null. All of these data are consistent with the model that Highwire and DFsn act together to form a functional ubiquitin ligase complex. In our model, this ligase complex targets Wallenda/DLK to restrain synaptic terminal growth, and an unknown substrate to promote synaptic function. We speculate that the targeting of the Wallenda/DLK MAPKKK by the Highwire/DFsn complex will be conserved from worms to mammals. While Highwire and DFsn collaborate for synaptic development, the male sterility of *DFsn *but not *highwire *mutants suggests that DFsn has Highwire-independent functions in other developmental processes.

## Conclusion

Here we demonstrate that the F-box protein DFsn binds to the RING-domain ubiquitin ligase Highwire and can localize to the *Drosophila *NMJ. DFsn is required to restrain synaptic growth and promote synaptic transmission. Genetic interactions between *DFsn *and *highwire *mutants indicate that *DFsn *and *highwire *function in the same pathway. Finally, DFsn, like Highwire, regulates the levels of the Wallenda/DLK kinase, and *wallenda *is necessary for both DFsn- and Highwire-dependent synaptic terminal overgrowth. These results support the model that Highwire and DFsn form a functional ubiquitin ligase complex that down-regulates the MAPKKK Wallenda/DLK to restrain synaptic terminal growth.

## Methods

### Tandem-affinity-purification procedure

Transgenic flies carrying the *UAS-NTAP-HiwΔRING *or *UAS-TAP *transgene driven by BG380 Gal4 driver were expanded. Fresh adult flies were deep frozen and then vortexed. Fly heads were then collected by passing the flies through sieves that separate the heads from other body parts. The purification procedure was performed at 4°C. Fly heads were homogenized with homogenization buffer (50 mM Tris-HCl pH 7.5, 125 mM NaCl, 5% Glycerol, 0.7% NP40, 1.5 mM MgCl_2_, 25 mM NaF, 1 mM EDTA, 0.2 mM DTT, 1 mM Na_3_VO_4_, 0.05 mM MG1115, 1 mM PMSF, Protease inhibitor cocktail (Roche Applied Science, Indianapolis, IN, USA). Two sequential centrifuge steps (17,000 rpm for 20 minutes and 45,000 rpm for 40 minutes) were used to clean the homogenate before loading the first column. The following purification steps were performed according to the Séraphin lab TAP protocol [25]. Final elutions from the second column were divided into two portions. The first portion was loaded to a SDS-PAGE gel (Criterion XT 4–12%) followed by Sypro Ruby staining. Individual bands were then excised, digested with trypsin, and subjected to LC-MS/MS. The second portion was precipitated, digested with trypsin and then subjected to LC-MS/MS. The searching program MASCOT (Matrix Science Inc., Boston, MA, USA) was used to identify the protein species. As a control, the TAP-only complex was analyzed by SDS-PAGE and no bands higher than 15 kDa were detected by Sypro Ruby staining.

### Fly stocks

Flies were maintained at 25°C on normal food. The following strains were used in this study: Canton S (wild type), P-f06595 (Exelixis Collection at Harvard Medical School), Df(2R)7872 (Bloomington Stock Centre), *elav *Gal4 (neuron specific) [26], BG380 Gal4 (neuron specific) [27], D42 Gal4 (motor neuron specific) [28], ELAV-GeneSwitch [29], *hiw*^*ND*51 ^and *hiw*^*ND*9 ^[3,12], two *wallenda *alleles: *wnd*^1 ^and *wnd*^3 ^[15].

### Transgenic constructs

Generation of TAP-tagged full-length *highwire *transgenes *UAS-NTAP-Hiw *and *UAS-NTAP-HiwΔRING *was described previously [11] using the TAP vector [20]. A *UAS-TAP *transgene was also generated for control purposes. A full-length cDNA clone (Clone ID: LD47425) for *DFsn *(CG4643) was obtained from Research Genetics (Huntsville, AL, USA). The entire coding region of *DFsn *was subcloned into pUAST-EGFP or pUAST-HM vector [30] to generate *UAS-GFP-DFsn *and *UAS-His-Myc-DFsn *transgenes. Transformant lines were generated using standard techniques.

### Immunoprecipitations and western blots

For immunoprecipitations, about 100 larval brains for each sample were collected by manual dissection. Larval brains were then homogenized in 0.5 ml lysis buffer (Tris-HCl 50 mM pH 7.4; NaCl 150 mM; EDTA 1 mM; NaF 2 mM; NP-40 0.2%; NaDOC (Na deoxycholate) 0.2% and protease inhibitors). Clean lysate was then incubated with the rabbit α-Highwire antibody [11] (HIW2b, 1:80), the mouse α-Myc antibody (9E10, 1:100), or the rabbit α-DVGLUT antibody [31] (1:80) for 2 hours. Protein A beads (25 ul; Roche) were added to the lysate. After overnight incubation, the beads were washed four times with lysis buffer before elution. HIW2b (1:250) and the mouse α-Myc antibody (1:1,000) were then used to probe the endogenous Highwire and the neuronally expressed Myc-DFsn (driven by BG380 Gal4), respectively, in the IP complex on western blots.

### Immunocytochemistry

Endogenous Wallenda was detected using the WndA1 antisera as described [15]. For all other antibodies, larvae were fixed in Bouin's solution (a 1:5:15 ratio of acetic acid/formalin/picric acid) for 8 minutes. The rabbit α-DVGLUT antibody was described previously [31], and used at 1:5,000 dilution. The α-fasciclin II (FasII; 1D4) antibody developed by Corey Goodman (Renovis, San Francisco, CA, USA) was obtained from the Developmental Studies Hybridoma Bank developed under the auspices of the NICHD and maintained by The University of Iowa, Department of Biological Sciences, Iowa City, IA, USA, and used at 1:5. To examine the localization of GFP-DFsn, GFP-DFsn was expressed in neuronal tissues by driving the *UAS-GFP-DFsn *transgene with ELAV-GeneSwitch in the presence of RU486. The mouse monoclonal α-GFP antibody (3E6; Invitrogen, Carlsbad, CA, USA) was then used at 1: 800 dilution to detect the GFP-DFsn protein.

### Imaging and analysis

Larvae were imaged on a Nikon (Tokyo, Japan) C1 confocal microscope. All images were acquired in third instar wild-type, mutant, and rescued larvae that had been simultaneously stained. The gain was chosen as the maximum gain that did not saturate the signal for each sample. A complete Z-stack was acquired for each NMJ or ventral nerve cord and rendered as a maximum projection. All quantifications were done at muscle 4 in segments A2–A4. The muscle sizes for all genotypes were similar. The number of type I boutons and the number of branch points of type I boutons were counted following α-DVGLUT and α-Fas II staining. A synaptic branch was defined as an arborization containing at least 2 type I boutons. Synaptic span was calculated by dividing the length of type I synapses by the length of muscle. The length of synapses and muscle were measured by an ocular micrometer (Nikon). Statistical analysis was performed and graphs were generated in Origin 7.0 (Origin Lab, Northampton, MA, USA). Each sample is compared to other samples in the group using ANOVA, with n for each condition described in the figure legends. All histograms are shown as mean ± standard error of the mean.

### Electrophysiology

Intracellular electrophysiological recordings were performed as described previously [32]. Both spontaneous mEJPs and evoked EJPs were recorded in HL-3 saline containing 0.47 mM Ca2+ [33]. At least 70 consecutive events were measured per cell using MiniAnal (Synaptosoft, Decatur, GA, USA) and averaged to determine the mean mEJP. Events with a slow rise time were rejected as artifacts from neighboring electrically coupled muscle cells. Evoked EJPs were recorded by stimulating the cut end of the segmental nerve with a 1 ms depolarizing pulse as described by Broadie [34]. Quantal content was estimated by dividing the mean EJP by the mean mEJP without correcting for non-linear summation, which would only have increased the observed differences.

## Competing interests

The author(s) declare that they have no competing interests.

## Authors' contributions

CW participated in the design of the study and performed all the experiments and analysis except for the electrophysiology. RWD performed the electrophysiological experiments. AD conceived the study, participated in its design and, together with CW, drafted the final manuscript, which was read and approved by all the authors.

## Supplementary Material

Additional file 1 Expression of a *DFsn *transgene in motoneurons rescues the morphological phenotype of *DFsn *mutants. **(a) **Representative confocal images of muscle 6/7 and muscle 4 synapses co-stained with DVGLUT (green) and FasII (red), in wild-type (WT), *DFsn *mutant [*P(f06595)/Df(2R)7872*] and *DFsn *motoneuron rescue (*DFsn*-MN Res) [*P(f06595)/Df(2R)7872;UAS-GFP-DFsn/D42-Gal4*] third instar larvae. **(b) **Quantification of bouton number of muscle 4 synapses in WT, *DFsn *and *DFsn *motoneuron rescue (*DFsn*-MN Res) third instar larvae (n = 22, 22 and 27 cells, respectively). The morphological defects in the *DFsn *mutant are rescued by the expression of GFP-DFsn in motoneurons (*p *< 0.001 for *DFsn*-MN Res versus *DFsn*; *p *> 0.9 for *DFsn*-MN Res versus WT). Click here for file
