# Supplementary figures and images for: DFsn collaborates with Highwire to down-regulate the Wallenda/DLK kinase and restrain synaptic terminal growth
Source: Neural Dev. 2007 Aug 15;2:16. doi: 10.1186/1749-8104-2-16 (PMC2031890; doi:10.1186/1749-8104-2-16)

A

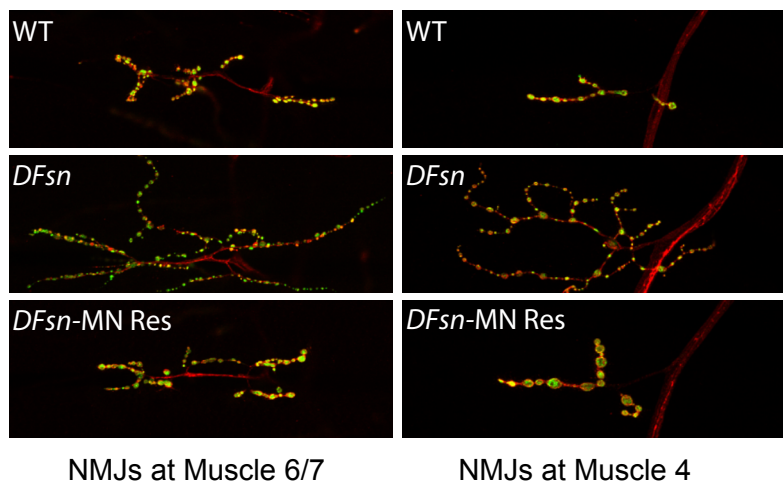

B

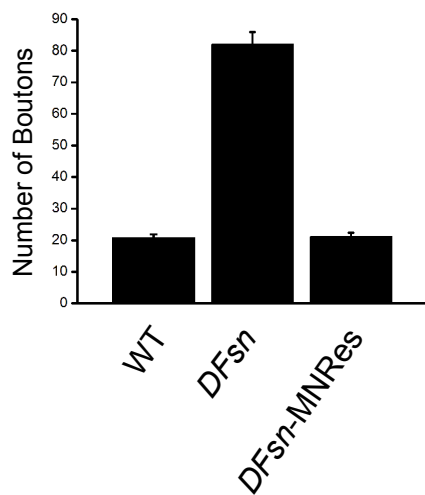

Supplement: Additional file 1 — Expression of a DFsn transgene in motoneurons rescues the morphological phenotype of DFsn mutants. (a) Representative confocal images of muscle 6/7 and muscle 4 synapses co-stained with DVGLUT (green) and FasII (red), in wild-type (WT), DFsn mutant [P(f06595)/Df(2R)7872] and DFsn motoneuron rescue (DFsn-MN Res) [P(f06595)/Df(2R)7872;UAS-GFP-DFsn/D42-Gal4] third instar larvae. (b) Quantification of bouton number of muscle 4 synapses in WT, DFsn and DFsn motoneuron rescue (DFsn-MN Res) third instar larvae (n = 22, 22 and 27 cells, respectively). The morphological defects in the DFsn mutant are rescued by the expression of GFP-DFsn in motoneurons (p < 0.001 for DFsn-MN Res versus DFsn; p > 0.9 for DFsn-MN Res versus WT). [file 1749-8104-2-16-S1.pdf]
